# Advancements in brain-computer interfaces for the rehabilitation of unilateral spatial neglect: a concise review

**DOI:** 10.3389/fnins.2024.1373377

**Published:** 2024-05-09

**Authors:** Alix Gouret, Solène Le Bars, Thibault Porssut, Florian Waszak, Sylvie Chokron

**Affiliations:** ^1^Integrative Neuroscience and Cognition Center (INCC), CNRS, Université Paris Cité, Paris, France; ^2^Research and Innovation Department, Capgemini Engineering, Paris, France; ^3^Institut de Neuropsychologie, Neurovision et Neurocognition (I3N), Fondation Ophtalmologique Adolphe de Rothschild, Paris, France

**Keywords:** brain-computer interface, unilateral spatial neglect, virtual reality, rehabilitation, serious game, neurotechnology

## Abstract

This short review examines recent advancements in neurotechnologies within the context of managing unilateral spatial neglect (USN), a common condition following stroke. Despite the success of brain-computer interfaces (BCIs) in restoring motor function, there is a notable absence of effective BCI devices for treating cerebral visual impairments, a prevalent consequence of brain lesions that significantly hinders rehabilitation. This review analyzes current non-invasive BCIs and technological solutions dedicated to cognitive rehabilitation, with a focus on visuo-attentional disorders. We emphasize the need for further research into the use of BCIs for managing cognitive impairments and propose a new potential solution for USN rehabilitation, by combining the clinical subtleties of this syndrome with the technological advancements made in the field of neurotechnologies.

## 1 Introduction

The recent and remarkable strides in bioengineering and artificial intelligence have substantially enhanced the field of non-invasive Brain-Computer Interfaces (BCIs) (Santamaría-Vázquez et al., 2023), spanning applications expanding from entertainment to healthcare (Douibi et al., 2021; Le Bars et al., 2021).

BCI technology enables the acquisition and translation of brain signals into digital commands that can be interpreted by external technological devices, providing an alternative to the typical “brain to peripheral nerves and muscles” information pathway (Fouad et al., 2015). As a result, BCIs have gradually entered the clinical area, in the form of rehabilitative devices—aiming to train or restore impaired cognitive and motor functions—or as assistive tools, allowing the compensation of the altered skills (Chaudhary et al., 2016). Considering such clinical applications of BCIs, it is noteworthy that particular emphasis has been placed on addressing motor and motion disabilities (Mane et al., 2020; Pichiorri and Mattia, 2020), due to their high and visible impact on patients’ daily life and to their high prevalence following various neurological diseases, such as stroke. In particular, despite its low spatial resolution, electroencephalography (EEG) has become one of the most popular noninvasive BCI for clinical use due to its low cost, direct measurement of brain activity and portability, making it a versatile and more acceptable tool relative to invasive methods (Nicolas-Alonso and Gomez-Gil, 2012).

In parallel, despite a rich literature on EEG markers associated with neurovisual and visuo-attentional processes (He et al., 2007; Zani, 2020), there is a noticeable lack of neurotechnological solutions and approaches enabling the management of deficits affecting this specific domain. Visuo-attentional impairments, such as unilateral spatial neglect (USN), are extremely frequent after brain lesions and can be particularly debilitating (Buxbaum et al., 2004; Spaccavento et al., 2019; Alnawmasi et al., 2022). However, these symptoms seem to fall within the spectrum of “invisible disability” (Thompson, 2019).

In this short review, we aim to pave the way for the consideration of state-of-the-art and non-invasive neurotechnology in the treatment of USN. We begin by describing the clinical and neurological specificities of USN. Subsequently, we review current non-invasive EEG-based BCIs and technological solutions dedicated to cognitive rehabilitation, with a particular focus on visuo-attentional disorders. Finally, we propose a potential new and relevant solution for treating USN by reconciling the clinical subtleties of this syndrome with the technological progress made in the BCI field.

## 2 Unilateral spatial neglect: general scope

### 2.1 Definition

USN is one of the most frequent disorders following a stroke. In fact, USN occurs in 25–30% of all stroke patients (Pedersen et al., 1997; Buxbaum et al., 2004; Esposito et al., 2021), approximately 50% of survivors of right hemisphere strokes (Buxbaum et al., 2004), and is typically associated with a poorer response to stroke rehabilitation and greater disability (Buxbaum et al., 2004; Chen et al., 2015; Spaccavento et al., 2017). USN is a complex syndrome clinically defined as “a failure to report, respond or orient to stimuli that are presented contralateral to a brain lesion, provided that this failure is not due to elementary sensory or motor disorders” (Heilman and Valenstein, 1979). This contrasts with other common disorders, such as hemianopsia, that consist in damaged visual function. Indeed, USN impacts not only visual perception but also attentional processes, thus affecting visuospatial awareness.

**Figure 1:**
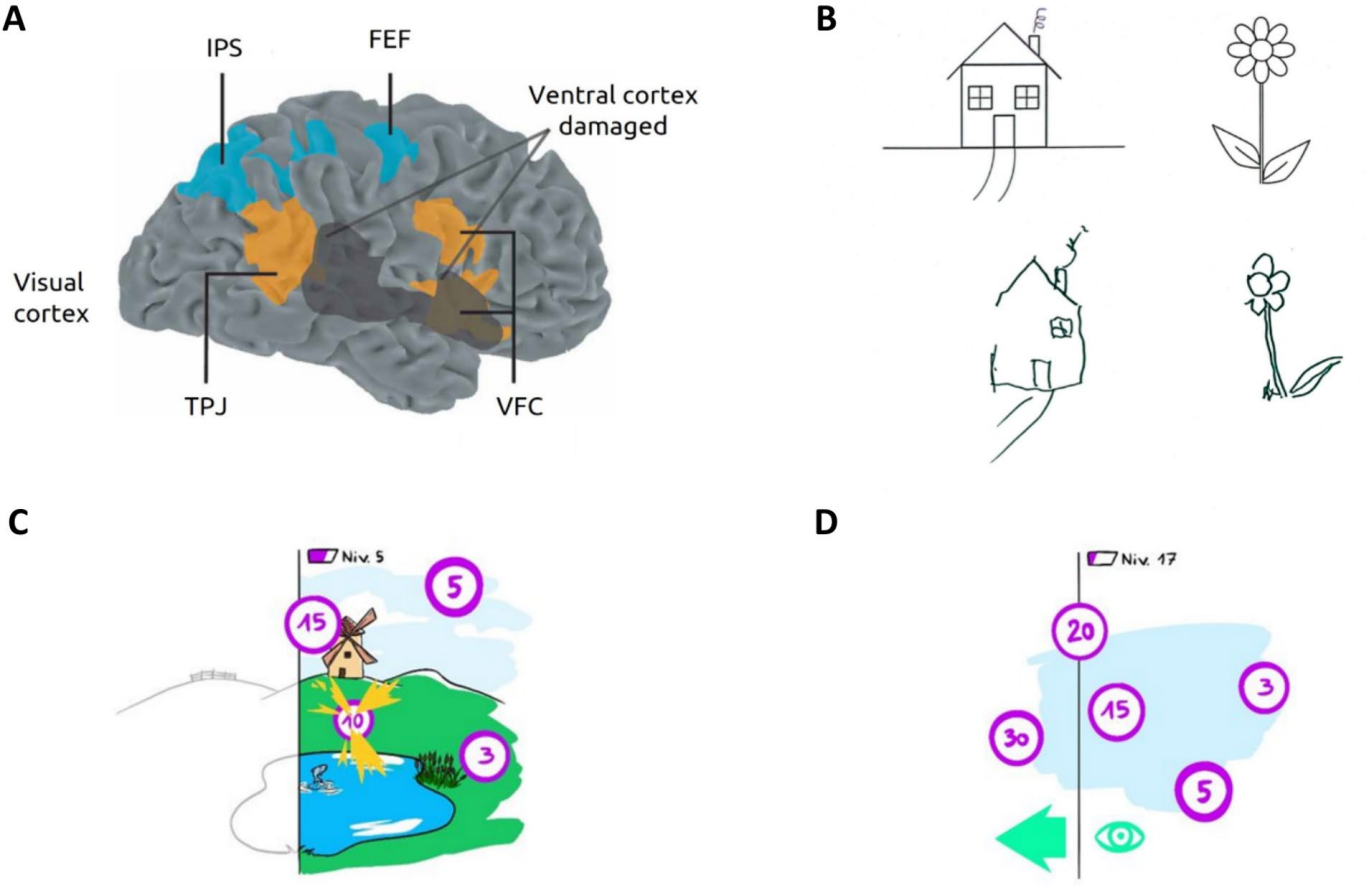
USN: illustration and rehabilitation prospect. **(A)** Human attentional networks involved in USN. The dorsal attentional network (DAN) in blue is involved in top-down processes; The ventral attentional network (VAN) in orange mediates bottom-up attentional processes. In black, hypothetical cortical lesion causing spatial neglect. FEF, frontal eye field; IPS, intraparietal sulcus; VFC, ventral frontal cortex; TPJ, temporoparietal junction. Adapted from Corbetta et al. (2005). The VAN and DAN rely on three important structures in visual attention processing: the FEF plays an important role in voluntary eyes movements; the right TPJ is notably involved in reorienting of attention toward unexpected or salient stimuli and the VFC is involved in unexpected / salient stimuli detection, reorienting attention toward them and facilitating responses. **(B)** Typical drawing copy produced by a patient with left USN, showing omissions of left-sided features from original images. **(C)** Illustration of the proposed visual scanning task based on VEP-paradigm and involving virtual reality to enhance USN rehabilitation. Patients are immersed in a playful virtual environment with flickering targets they must find and catch with their attention to win points and progress. The patient’s neglected hemifield is represented in black and white. **(D)** An asymmetric rewards principle is employed, whereby the most rewarding targets are closer to the patient’s neglected visual field (i.e., left) encouraging them to explore their contralesional space by establishing operant conditioning.

USN can manifest in various ways, leading to the classification of different subtypes of hemineglect, each associated with specific brain lesion sites (Rode et al., 2017), predominantly in the frontal and parietal areas (Corbetta et al., 2005) (Figure 1A). Symptoms can be categorized based on the reference frame (egocentric or allocentric, i.e., object-centered neglect irrespective of its position relative to the person), modality (motor, perceptual, intentional, or representational neglect) and the sector of space involved, including imaginary space (personal, peri-personal and extrapersonal) (Buxbaum et al., 2004; Rode et al., 2017; Gammeri et al., 2020). Near and far space neglect can also be distinguished. The resulting deficit in attention and awareness of one hemispace significantly impacts patients’ behavior and impedes overall stroke rehabilitation, particularly after right brain damage (RBD) (Stone et al., 1991; Esposito et al., 2021).

Spontaneous recovery from hemineglect can occur within the first 3 months post-stroke. However, more than one-third of USN patients show chronic symptoms after one year, especially following RBD (Nijboer et al., 2013). Given the widespread impact of USN on daily life, this syndrome necessitates careful attention and management.

### 2.2 Laterality and attentional mechanisms involved in USN

Right and left USN appear equally common after left and right brain damage during the acute phase. However, left USN after RBD tends to be more noticeable, enduring and severe (Rode et al., 2017). Although the occurrence of hemineglect varies among studies based on experimental protocols, left USN predominates in the later stages (Beis et al., 2004; Buxbaum et al., 2004; Esposito et al., 2021).

Underlying mechanisms related to the right hemisphere’s specialization for spatial attention and awareness (Heilman and Valenstein, 1979) could explain the lateralized and heterogeneous nature of symptoms.

USN has been explained as both a deficit in orienting attention to the contralesional hemispace (Heilman and Valenstein, 1979) and a pathological hyper-attention to the ipsilesional hemispace (see Bartolomeo and Chokron, 2002 for review).

Two distributed cerebral networks control different components of attention (Corbetta and Shulman, 2002). The dorsal attentional network (DAN) serves as a top-down system involved in stimuli selection, saccadic scanning eyes movements, exogenous and spatial attention. The DAN exhibits bilateral frontoparietal networks interactivity, particularly between the frontal eye fields and intraparietal sulcus that compose it (Corbetta et al., 2005; Corbetta and Shulman, 2011) (Figure 1A). On the other hand, the ventral attentional network (VAN) operates as a stimulus-driven (bottom-up) system regulating endogenous attention, reorientation of attention, vigilance, and response to alertness. The VAN mainly relies on the right temporoparietal junction and the ventral frontal cortex (Husain and Rorden, 2003; He et al., 2007; Corbetta and Shulman, 2011; Bartolomeo et al., 2012) (Figure 1A). While functionally distinct, previous research suggests that both networks interact and work together in certain attentional processes (Husain and Rorden, 2003; He et al., 2007; Corbetta and Shulman, 2011; Bartolomeo et al., 2012).

These distributed cortical networks are disrupted in USN (Corbetta and Shulman, 2002; Bartolomeo et al., 2012). Their dysfunction could better explain USN complex symptoms than specific structural changes at the lesions sites (e.g., intrahemispheric disconnections) (Buxbaum et al., 2004; Corbetta et al., 2005). Impaired functionality of the VAN could indirectly cause dysfunction of the bilateral DAN (He et al., 2007), explaining the higher severity of USN caused by RBD. This vision is supported by the recovery mechanisms and reorganization of attentional networks observed during rehabilitation (Corbetta et al., 2005; Umarova et al., 2016).

### 2.3 USN evaluation and management—current approaches

The current gold standard for clinical USN assessment is the behavioral inattention test (BIT) which employs a brief battery of pen-and-paper screening tests to determine the presence, extent and nature of neglect in daily life situations (Wilson et al., 1987). Such tests include cancelation tasks, visual search, copying and representational drawing (see Figure 1B), as well as visual extinction assessment. However, no consensus exist on their efficiency to diagnose USN given the challenges in detecting and identifying underlying types of motor and visual neglect (Williams et al., 2021). Numerous developments have explored the use of computerized assessment tasks (notably involving virtual reality) (Ogourtsova et al., 2018) to address the limitations of existing methods (Giannakou et al., 2022), such as their lack of ecological validity and inability to detect compensatory strategies (Azouvi, 2017; Williams et al., 2021). In particular, standard pen-and-paper tests batteries have been translated to VR with comparable or improved performance, and new USN management platforms have emerged (Fordell et al., 2016; Knobel et al., 2020).

Therapeutic approaches for USN rehabilitation can be categorized into bottom-up and top-down strategies, primarily contrasting visual scanning training (VST) with methods involving aids such as prism adaptation (PA; Gammeri et al., 2023). While the top-down approach consists in teaching the patient compensatory strategies for neglect (with short-term impact), the bottom-up approach aims to remediate attention biases and spatial representations. Typically, VST promotes neuroplasticity and the reorganization of disrupted attentional networks by encouraging active exploration of the environment. On the other hand, PA induces an artificial visual shift to the neglected space, temporarily modifying sensorimotor mapping. Mental practice has also been used to improve the perception of the contralesional limbs in neglect patients. Such task consists in imaging performing a movement, for instance with the contralesional upper limb to improve patients’ self and spatial perception (Welfringer et al., 2011). Other approaches involving brain stimulations or robot-assisted motor rehabilitation exist (see Durfee and Hillis, 2023; Singh and Leff, 2023 for reviews) with no clear consensus on their efficacy. Amidst the growing interest in computer-based therapy, the implementation of VR protocols emerges as a promising intervention option for USN therapy (Gamito et al., 2017; Durfee and Hillis, 2023). Notably, VR provides enriched rehabilitation possibilities, including multimodal stimulations, immersiveness, ecological training conditions, and the ability to implement various rehabilitation strategies (Kim et al., 2011; Van Kessel et al., 2013). Several innovative rehabilitative methods for USN now rely on VR (Yasuda et al., 2017; Ogourtsova et al., 2018; Huygelier et al., 2020; Knobel et al., 2021). Compared to conventional rehabilitation programs, immersive VR-based training usually improves patients adherence and compliance with treatment (Martino Cinnera et al., 2022).

Surprisingly, despite substantial advancements in neurotechnologies, the application of rehabilitative BCIs within the context of USN remains largely unexplored. In the following section, we will delve into the realm of non-invasive BCI technology and its untapped potential.

## 3 BCIs potential for stroke and cognitive rehabilitation

### 3.1 BCIs categories and definition

BCIs are primarily designed to replace, restore, enhance, supplement, or improve cognitive and motor functions (Daly and Huggins, 2015). This technology holds significant potential for stroke rehabilitation, as BCIs can promote neuroplasticity through various mechanisms and have proven efficiency in providing communication channels, restoring or compensating motor functions loss in patients (Zander et al., 2014; Mane et al., 2020).

Among clinical applications, two types of BCIs have been developed. Assistive BCIs aim to bypass damaged brain pathways and compensate the deficits by providing alternative means for impaired individuals to interact with their environment. Rehabilitative BCIs, on the other hand, aim to stimulate the recovery of the damaged neural networks within the brain, thereby facilitating the restoration or relearning of lost functions (Mane et al., 2020). The latter approach has demonstrated its efficiency to tackle post-stroke syndromes, particularly contralesional motor deficits (Chaudhary et al., 2016).

These two approaches are based on distinct BCI paradigms (see Table 1) that reflect both the mental activities performed by the participants and the brain signals used to construct the interface. They are thus categorized as passive, active and reactive BCIs (Zander et al., 2014). Clinical BCIs primarily involve active and reactive paradigms. Therefore, we will focus on these two categories in the remainder of this section.

**Table 1:** Examples of non-invasive BCIs applications in both assistive and rehabilitative contexts.

BCI paradigm	Assistive application	Rehabilitative application
MI (active BCI)	Device control: exoskeleton etc. (Ferrero et al., 2023)	Prepare physiotherapy and promote motor recovery (Barsotti et al., 2015; Barria et al., 2021; Guo et al., 2022)
NF (active BCI)		Enhance cognitive functions (Zoefel et al., 2011; Enriquez-Geppert et al., 2017) Improve attention-related disorders (Gevensleben et al., 2009; Saj et al., 2018; Moradi et al., 2024)
P300 (reactive BCI)	Communication (spellers) (Amiri et al., 2013) Device control (Lopes et al., 2013)	
SSVEP/c-VEP (reactive BCI)	Communication (Gembler et al., 2020; Mannan et al., 2020) Device control (Kwak et al., 2015)	Promote motor recovery (Guo et al., 2022) Improve attentional impairments (Arpaia et al., 2020)

MI, motor imagery; NF, neurofeedback; SSVEP, steady-state visually-evoked potential; c-VEP, code-modulated visually-evoked potential.

Active BCIs involve intentional modulation of brain activity, often through motor imagery (MI-BCI), which relies on the mental execution of movements without muscular activation. MI-BCIs are currently the dominant type on BCI-based post-stroke motor-related rehabilitation (Mane et al., 2020) and promote motor recovery by increasing functional activity in damaged motor brain regions involved in intended movements execution (Chaudhary et al., 2016; Mane et al., 2022).

Active BCIs also include NF-BCIs in which users learn to self-regulate brain activity or improve cognitive functions by getting real-time feedbacks. For example, they may train to increase attention-related brainwave activity, receiving immediate feedback of their performance like visual or auditory cues (Zoefel et al., 2011).

Reactive BCIs rely on specific cerebral responses to external stimuli, such as visually-evoked potential (VEP) or P300 event-related potential, to facilitate communication or device control (Amiri et al., 2013). P300 is typically triggered by the apparition of a rare or desired stimulus among non-targets. It is commonly used in spelling device, where users focus on symbols arranged in a grid, with the system detecting the P300 when the desired symbol is flashed. VEP-based BCIs (VEP-BCIs) require the user to focus their attention on stimuli that exhibit either periodic (steady-state VEPs, SSVEPs) or pseudo-periodic (code-modulated VEPs, c-VEPs) repetitive behavior, such as high-contrast flickering (Herrmann, 2001; Martínez-Cagigal et al., 2021). The flicker frequency or pattern is then mirrored through brain activity, enabling to select or discriminate a specific target among stimuli. VEP-BCIs typical assistive applications are spellers, exoskeleton control etc. (Bin et al., 2011; Kwak et al., 2015; Mannan et al., 2020). Henceforth, the term VEP will refer to both SSVEP and c-VEP.

### 3.2 BCIs in post-stroke rehabilitation

Stroke can result in a wide range of impairments, including motor, cognitive and attentional deficits. However, the field of BCIs for poststroke cognitive rehabilitation remains quite nascent and has received significantly less attention from researchers compared to motor neurorehabilitation, despite being a key factor in overall stroke recovery and outcomes (Barker-Collo et al., 2010; Mane et al., 2020, 2022).

Indeed, after brain damage, motor function restoration is usually the primary focus. Many MI-BCIs have been successfully developed to this end. For example, MI-BCIs enable patients to control prosthetics and perform voluntary movements, by imaging their execution, stimulating neural plasticity and strengthening associated neural networks (Case et al., 2015). Barria et al. (2021) introduced a promising MI-BCI for post-stroke patients with lower limb hemiparesis to regain ankle mobility. Patients were instructed to imagine moving or relaxing their foot, receiving feedback from an ankle skeleton that facilitated dorsiflexion or relaxation accordingly.

Until recently, only NF-BCI paradigm had been applied to poststroke cognitive rehabilitation (Carelli et al., 2017; Mane et al., 2022). For instance, this paradigm enabled to enhance cognitive functions (Zoefel et al., 2011; for review see Enriquez-Geppert et al., 2017) and improve various conditions, including neurodevelopmental and neurodegenerative disorders—such as attention-deficit/hyperactivity disorders (Gevensleben et al., 2009) or aphasia in stroke patients (Ferro et al., 2004).

Although BCIs have demonstrated effective recovery and may promote neuroplasticity, no efficient BCI device has yet been proposed to manage cerebral visual impairments resulting from brain lesions.

### 3.3 BCI and USN

#### 3.3.1 USN challenges in BCI implementation

While recent enthusiasm surrounds computer-based USN therapy with promising results (Cavedoni et al., 2022; Giannakou et al., 2022), further research into BCI technology potential in this field is still needed. Recent reviews (Durfee and Hillis, 2023; Singh and Leff, 2023) examining modern technologies and current methods for USN management mention the increasing interest in neurotechnologies, such as augmented and virtual reality, but do not delve into BCI-based interventions. There is limited research available on the implementation of BCIs in USN rehabilitation, with only two studies by Saj et al. (2018, 2021) that tested such applications in USN patients. The authors introduced an EEG-based NF-BCI to improve USN recovery. In this intervention, patients were instructed to lower their alpha amplitude, with feedback provided on a computer screen reflecting the variation. For instance, successfully reducing alpha amplitude resulted in a spaceship moving forward, demonstrating promising results.

Interestingly, certain BCI paradigms have already shown potential in USN management, notably Motor Imagery and Visually Evoked Potentials. For instance, visuomotor imagery helped improving the perception of the contralesional upper limb and reduced USN deficits in subacute neglect patients (Welfringer et al., 2011). Furthermore, BCI-like tools based on VEP analysis and spectral characteristics, such as VEP latency in USN patients (Spinelli et al., 1994), have been developed to detect and assess the degree of USN in patients. Recently, Mak et al. (2022) proposed an EEG-guided detection device involving augmented reality based on bandpower / spectrospatial features analysis to detect USN and map the estimated neglect visual field. Integrating BCI technology into these applications could improve their effectiveness, as patients would receive real-time feedback on their performance, leading to more interactive devices that could enhance their engagement to therapy. However, it is worth noting that no BCI have been specifically designed for the management of visuo-attentional impairments.

Challenges in BCI implementation may contribute to the lack of consideration for BCI-based cognitive rehabilitation in USN, including issues such as BCI illiteracy, complexity, and interfaces ergonomics.

BCI illiteracy refers to a lack of proficiency in using a BCI system under standard training conditions (Volosyak et al., 2020), particularly evident in MI-BCIs. MI-BCIs heavily rely on individual characteristics: inter-subject variability may significantly impact BCI performance and the ability to perform MI tasks. While research efforts have been made to improve MI-BCIs design and mitigate BCI illiteracy (Jeunet et al., 2016), addressing this phenomenon remains a major challenge in advancing BCI technology (Thompson, 2019).

The lack of ecological validity in BCI systems poses challenges in usability and devices development. The typical feedback and stimuli used can be fatiguing, affecting the overall user experience and decreasing BCI performance. To develop applications suitable for patients, a trade-off must be found between BCI performance and user experience (Yoshimoto et al., 2017), especially in neurorehabilitation BCIs.

#### 3.3.2 What VEP-based BCI could provide for USN

In the realm of non-invasive reactive BCIs, VEP-BCIs have undergone extensive study in recent years (Vialatte et al., 2010; Duart et al., 2020). Notably, this paradigm has been clinically applied in the rehabilitation of neurodevelopmental attentional disorders (Arpaia et al., 2020) and the advancement of communication devices (Chaudhary et al., 2016; Mannan et al., 2020). This neurotechnology seems well-suited to enrich and benefit USN management.

The VEP paradigm is notable for its ability to measure an individual’s state of attention or awareness, given the strong correlation between concentration level and VEPs amplitude (Morgan et al., 1996; Herrmann, 2001; Kashiwase et al., 2012). Traditional bottom-up cognitive rehabilitation for USN precisely seeks to retrain attention engagement and orientation toward the neglected space, aiming to restore awareness of stimuli presented contralesionally. BCI-based interventions requiring sustained attention to specific stimulations, such as VEP-BCIs, could then provide a reliable approach for USN management (Van Vleet and DeGutis, 2013), as they enable the monitoring of awareness and consciousness level to stimuli. This monitoring is not achievable with other technologies that solely rely on behavioral indicators, such as eye-tracking. This specificity combined with feedback on user behavior (e.g., focus on a target leads to its validation and winning points) fosters a deeper connection between user intentions and outcomes, thereby cultivating a greater sense of agency over the attentional process (Nierula et al., 2021).

Regarding attentional demands, some BCI paradigms, such as MI or NF, require high energy and concentration levels, which may be incompatible for individuals with certain neurological conditions. Conversely, VEP-BCIs seem to improve user experience, training responsiveness and overall performance in comparable tasks (Guo et al., 2022). The calibration process for SSVEP-BCIs is generally shorter compared to MI and NF paradigms but can vary depending on the number of available classes and specific application requirements, sometimes resulting in lengthy calibration periods. Recent advancements in VEP-BCIs, particularly c-VEPs, enable multi-class scenarios while maintaining rapid calibration (Martínez-Cagigal et al., 2021; Cabrera Castillos et al., 2023).

Additionally, VEP-BCI paradigm shows a lower BCI illiteracy rate and better robustness to inter-individual variability (Lee et al., 2019; Volosyak et al., 2020).

Due to potential increased sensitivity to brightness among brain damage individuals, ongoing VEP research aims to alleviate visual discomfort and fatigue associated with this paradigm, which currently limit its application (Chai et al., 2020). Strategies include incorporating movement-based periodic behaviors to elicit VEPs (Yan et al., 2017; Auda et al., 2022), reducing flickering contrast (Ladouce et al., 2022), adapting the shape of the stimuli (Duart et al., 2020), and exploring other stimulation features (Armengol-Urpi and Sarma, 2018; Chen et al., 2019). This research aligns with the imperative to mitigate potential risks associated with epileptogenic frequencies, a critical consideration in VEP-BCIs due to the use of highly contrasted flickering stimuli.

Finally, USN is the result of brain lesions with diverse typology (see Figure 1A). However, VEPs recorded via EEG have the interesting characteristic of being particularly prominent in the occipital region (Cabrera Castillos et al., 2023; Reitelbach and Oyibo, 2024), the deterioration of which is generally not associated with USN. Therefore, the setup and accuracies of c-VEP/SSVEP-BCI in USN patients should remain relatively stable.

## 4 Discussion and prospects in non-invasive BCI-based cognitive rehabilitation for USN

### 4.1 Promising neurotechnologies for USN

To date, no effective BCI device has been proposed for the treatment of cerebral visual impairments, which are prevalent following stroke and hamper overall rehabilitation effectiveness. Despite the acknowledged impact of BCIs on neuroplasticity for motor recovery following brain injury, there remains a scarcity of applications targeting cognitive and attentional deficits. Nevertheless, through the current review, we identified two technologies particularly promising for USN management: VEP-BCIs and VR.

VEP-BCIs demonstrate particular accuracy in measuring attentional and awareness levels (Kashiwase et al., 2012), and have already shown interesting results in addressing neurodevelopmental attentional disorders (Arpaia et al., 2020). Implementing VEP-based paradigm within selection tasks, such as visual scanning training (VST), could enhance USN management. Indeed, this hybrid approach would effectively combine both bottom-up and top-down rehabilitation strategies, by encouraging patients to search for targets, redirecting attention to the contralesional hemispace, and requiring sustained attention, thus stimulating the disrupted attentional networks (see Figure 1A).

As mentioned in section 2.3, VR-based USN therapy incorporating attention training and VST has received considerable attention for its ability to augment standard rehabilitation. VEP-BCI interest could be further enhanced if associated with ecological environment featuring standardized and well-integrated stimuli, which aligns with current research emphasis. In this context, the combination with VR technology could address the limitations of current rehabilitative methods, by providing 3D, naturalistic, and fully controlled environments. VEP-BCI could complement these developments by enabling precise monitoring of attentional processes and removing motor control requirements.

### 4.2 Prospects for USN rehabilitation combining VEP-BCI and VR

Integrating BCI into VR-based cognitive rehabilitation appears promising for addressing specific nuances of USN, by providing precise and adaptative interventions that would dynamically address patients’ individual needs. A VST application combining VR and VEP-BCI (VR-VEP-BCI) would harness the benefits of both technologies, simultaneously training a broad spectrum of attentional processes disrupted in USN, mirroring real-life conditions. VR facilitates multimodal stimulations, allowing for exogenous attention training through the use of multisensory cues to redirect attention to the contralesional hemispace. This challenges the spatial awareness of USN patients, while enhancing VST efficacy (Fordell et al., 2016). Additionally, sustained attention would be necessary for target detection validation and in device interaction, engaging endogenous attention as well.

Typically, in the proposed approach, USN patients would be immersed in a virtual environment with flickering targets to catch by focusing their attention on them to earn points and progress in the game for explicit rehabilitation (Figure 1C). With an asymmetric reward system, where the most rewarding targets are predominantly located near the neglected hemifield for increased difficulty, patients would be implicitly encouraged to explore the ipsilesional space through operant conditioning mechanisms (Figure 1D). Finally, the VEP paradigm establishes a direct link between gaze and conscious perception, which is not achievable with other technologies, such as eye-tracking. This direct link creates a strong sense of agency (Nierula et al., 2021) and enhances the overall effectiveness of the rehabilitation approach.

Despite the potential of VR-VEP-BCI for USN cognitive rehabilitation, challenges remain in overcoming hardware limitations and ensuring efficient BCI, while maintaining immersion and a comfortable VR experience.

### 4.3 Conclusion

Combining advanced interventions with traditional therapeutic protocols may be the future of USN rehabilitation, enriching the possibilities and mixing strategies, toward a holistic approach. VR-BCI combination not only offers interesting perspectives for USN management but also for enriching cognitive models of attention orientation in space with this particularly adapted pathological model. Integrating cognitive therapy into existing BCI-based motor therapies could synergically enhance the overall effectiveness of post-stroke USN rehabilitation programs, addressing the heterogeneous nature of USN. Further research and investigations are now required to draw firm conclusions about the clinical efficacy of the latest approaches.

## Author contributions

AG: Writing – original draft, Writing – review & editing. SL: Supervision, Writing – original draft, Writing – review & editing. TP: Writing – review & editing. FW: Supervision, Writing – review & editing. SC: Supervision, Writing – review & editing, Validation.
